# An Effective SARS-CoV-2 Electrochemical Biosensor with Modifiable Dual Probes Using a Modified Screen-Printed Carbon Electrode

**DOI:** 10.3390/mi12101171

**Published:** 2021-09-29

**Authors:** Sow-Neng Pang, Yu-Lun Lin, Kai-Jie Yu, Yueh-Er Chiou, Wai-Hung Leung, Wen-Hui Weng

**Affiliations:** 1 Department of General Medicine, Mater Misericordiae University Hospital, 351402 Dublin, Ireland; benpang2004@gmail.com; 2 Department of Chemical Engineering and Biotechnology, Graduate Institute of Biochemical and Biomedical Engineering, National Taipei University of Technology, Taipei City 106, Taiwan; 11xc11860324@gmail.com (Y.-L.L.); m7398@cgmh.org.tw (K.-J.Y.); 3 Division of Urology, Department of Surgery, Linkou Chang Gung Memorial Hospital, Taoyuan 33305, Taiwan; 4 College of Medicine, Chang Gung University, Taoyuan 333, Taiwan; 5 Department of Nursing, College of Medicine, Fu Jen Catholic University, New Taipei City 242, Taiwan; fjunurse1990@gmail.com; 6 Division of Colorectal Surgery, Department of Surgery, Mackay Memorial Hospital, Taipei City 104, Taiwan

**Keywords:** SPCE biosensor, COVID-19, SARS-CoV-2

## Abstract

Due to the severe acute respiratory syndrome coronavirus (SARS-CoV-2, also called coronavirus disease 2019 (COVID-19)) pandemic starting in early 2020, all social activities ceased in order to combat its high transmission rate. Since vaccination combats one aspect for halting the spread of the virus, the biosensor community has looked at another aspect of reducing the burden of the COVID-19 pandemic on society by developing biosensors that incorporate point-of-care (POC) testing and the rapid identification of those affected in order to deploy appropriate measures. In this study, we aim first to propose a screen-printed carbon electrode (SPCE)-based electrochemical biosensor that meets the ASSURED criteria (i.e., affordable, sensitive, specific, user-friendly, rapid, equipment-free, and deliverable) for POC testing, but more importantly, we describe the novelty of our biosensor’s modifiability that uses custom dual probes made from target nucleic acid sequences. Additionally, regarding the sensitivity of the biosensor, the lowest sample concentration was 10 pM (*p* = 0.0257) without amplification, which might challenge the traditional technique of reverse transcriptase-polymerase chain reaction (RT-PCR). The purpose of this study is to develop a means of diagnostics for the current pandemic as well as to provide an established POC platform for future epidemics.

## 1. Introduction

SARS-CoV-2 was declared a global pandemic in early 2020, and since then, the world has been in lockdown [1]. We understand that the zoonotic virus SARS-CoV-2 is an enveloped single-stranded RNA that has been identified to have 29,881 base pairs, encoding 10 genes, and is classified within the Betacoronavirus genus [2]. The new virus has been identified to share sequence homology with viruses responsible for previous outbreaks, including SARS-CoV-2 and Middle East respiratory syndrome coronavirus (MERS-CoV) [2,3,4].

In response to the outbreak, the World Health Organization (WHO) has announced multiple guidelines over the year with a few updates along the way; however, at its core, the essentiality has remained unchanged [5]. To achieve the goal of maintaining a low level of transmission, the WHO has outlined six essential criteria—one of which includes testing [6]. Currently, the gold standard for SARS-CoV-2 testing is reverse transcriptase polymerase chain reaction (RT-PCR) [7], as outlined by many laboratories and the WHO [8,9].

Over the past year, as a result of this pandemic, numerous studies regarding the detection of SARS-CoV-2 using electrochemical biosensors have emerged with promising prospects. The reason for the rise in these biosensors is not a mystery, as the pitfall of RT-PCR is that it is a time-consuming process, involving transferring samples to a central laboratory, preparation, and, on average, 2 to 4 h of processing time [8,9]. Moreover, there is a likelihood of contamination of samples producing false positives [10]. To date, many studies have developed biosensors targeting SARS-CoV-2 antigens [11,12]; however, few studies have looked at electrochemical detection of nucleic acid sequences [13]. Our design for the biosensor originated from a recent study involving the detection of miRNA in breast cancer and colorectal cancer patients’ urine [14], hence, our focus in SARS-CoV-2 nucleic acid detection.

In this study, we adopt the same technology with modifications by using an established SPCE platform with surface modifications and dual probe sensors, with changes to the probes, the FITC detector probe, and the biotinylated capture probe, specific to that of the SARS-CoV-2 RNA nucleotide sequence, which is then functionalized following HRP and H_2_O_2_ redox reactions with TMB substrate, and subsequently detected by cyclical voltammetry (CV), linear sweep voltammetry (LSV), and chronoamperometry (CA). The aforementioned biosensor is designed to adapt to the targeted nucleic acid sequence and to have a processing time of less than an hour with minimal handling. It is hoped that it would improve point-of-care (POC) testing and management in emergency departments and in community settings such as general practices and family practices, and, therefore, aid in the efforts of the current pandemic, and also allow rapid access to testing in future outbreaks.

## 2. Materials and Methods

The design for this study was remodeled after a previous study produced by our laboratory by using a modified SPCE and dual probes for the detection of microRNAs in select patients’ urine that has shown promising results [14]. Accordingly, it should be possible to use a similar concept for detecting SARS-CoV-2 RNA specific sequences from patients’ saliva/oropharyngeal swab, serum, or urine [15]. Hence, minimal adjustments to the experimental protocol were required. The materials and methods are described briefly in the following sections.

### 2.1. Reagents and Chemicals

In the current study, the materials required for the SPCE’s sensor platform were based on Leung et al., 2021 with minor modifications [14]. A summary of the materials that were applied in this study includes: CM-dextran sodium salt (CMD-Na), phosphate-buffered saline (PBS), 1-ethyl-3-(3-dimethylaminopropyl) carbodiimide (EDC), N-hydroxysulfosuccinimide (NHS), ethanolamine, potassium chloride (KCl), potassium ferricyanide (Fe(CN)63−), hydrogen peroxide (H2O2), diethyl pyrocarbonate (DEPC), and 3,3′,5,5′-tetramethylbenzidine (TMB) purchased from Sigma-Aldrich (St. Louis, MO, USA). Anti-fluorescein horseradish peroxidase (HRP), a 40,000 dalton protein, was supplied by Abcam (Cambridge, UK). MES free acid monohydrate, hydroxymethyl-aminomethane (Tris), and ethylenediaminetetraacetic acid (EDTA) were purchased from Amresco Inc. (Solon, OH, USA). Core streptavidin was from BiVision (Bioptics, Tucson, AZ, USA) and sodium chloride (NaCl) was from Promega Corporation (Madison, WI, USA). The screen-printed carbon electrodes (SPCEs) were purchased from Zensor R&D (Taichung, Taiwan).

### 2.2. FITC Detector Probe and Biotinylated Capture Probe Designs

At the time of this study, Taiwan was not experiencing surges of COVID-19 cases; therefore, the acquisition of real collected samples was not possible. Therefore, the mimic COVID-19 case sequence was designed according to the NCBI SARS-CoV-2 database and named as Target: 5′-GCG CGA CAT TCC GAA CGC TGA AGC GCT GGG GGC AAA TTG T-3′. The dual probes that were designed for detecting and capturing the signals from the sensor were named the biotinylated probe (capture probe) (5′-TTC TTC GGA ATG TCG CGC-3′-biotin) and the fluorescein (FITC) ssDNA probe (detector probe) (fluorescein -5′ACA ATT TGC CCC CAG CGC TTC AG -3′). Moreover, the unspecific single nucleus RNA sequences were named miR-21 (5′-UAG CUU AUC AGA CUG AUG UUG A-3′) and miR-141 (5′-UAA CAC UGU CUG GUA AAG AUG G-3′) and were both applied to test the selectivity experiments; all the sequences were designed by ourselves and synthesized by Genomics (Genomics, Taiwan, Taipei).

### 2.3. Surface Modification of SPCE

In order to ensure that the SPCE could bind the biotinylated capture probe more stably with working electrode efficiency, according to our previously report [14], in brief, first we introduced a carboxylic (–COOH) functional group onto the carbon electrode surface, and 50 μL carboxymethyldextran sodium salt (CMD- Na) (50 mg/mL) was used to saturate the work surface for 16 h, and then a prepared mixture of 8 mg/mL 1-ethyl-3-(3-dimethylaminopropyl) carbodiimide (EDC) and 22 mg/mL N-hydroxysulfosuccinimide (NHS) in 0.1 M MES buffer (pH 4.7) was used for 15 min at room temperature. This allowed the leading streptavidin (BioVision Inc. Mountain View, CA, USA) to conjugate on the surface. Finally, we immobilized the 5 μM biotinylated ssDNA probe that contained the partially mimicked COVID-19 case sequence and 1 M ethanolamine to block the remaining activated sites. This formed the functional surface of the “bioreceptor” that complemented the corresponding end sequence of the target probe. The experimental solutions were all configured using 0.1% DEPC-treated water to inactivate RNase and to ensure the stability of the reactions. Then, scanning electron microscopy (SEM, HITACHI S3000H, Tokyo, Japan) was performed to confirm the dripped into 0.4 mM of 3,3′,5,5′-tetramethylbenzidine (TMB) and 0.4 mM of H2O2 suitability of the surface of the SPCE after completing all the steps.

### 2.4. Reactions among Targeted Sequences, the Biotinylated Capture Probe, and the FITC Detector Probe

**Figure 1:**
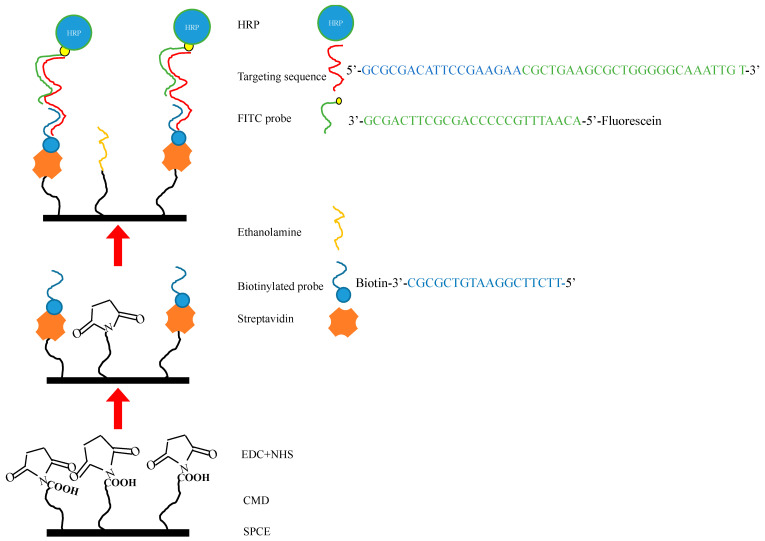
Establishment of the SARS-CoV-2 biosensor using the modified screen-printed carbon electrode (SPCE) and probe design. CMD was used to modify the SPCE to provide the reactive groups, followed by EDC and NHS crosslinking to the electrode. Streptavidin was subsequently added to the electrode. After this, the biotinylated probe was immobilized onto streptavidin, with ethanolamine occupying the remaining SPCE surface. In order to detect the mimicked targeting sequence, pretreatment of RNA and the fluorescein-modified detection probe (FITC probe) was conducted in a separate medium. The addition of the targeting sequence/FITC probe complex to the functional SPCE surface allowed for hybridization. With the addition of anti-fluorescein horseradish peroxidase (HRP), a catalyzed redox reaction occurs between tetramethylbenzidine (TMB) and H_2_O_2_ at the surface of the electrode.

There were four main processes before signal detection. The FITC detector probe was initially mixed with mimic COVID-19 sample sequences, then, instilled with 7 μL DEPC-treated water, 2 μL 1× STE buffer (0.1M NaCl,10 mM Tris, 1 mM EDTA, pH 8.0) at 68 °C for 3 min, and subsequently cooled to 4 °C for 15 min. The procedures were all performed without light. This process marked the completion of the initial preparation to allow SARS-CoV-2 target sequence hybridization with the modified SPCE, which occurred by direct application to the working surface for 15 min at room temperature. After this, 20 μL of anti-fluorescein antibody (HRP) (Abcam) was applied by dripping for 15 min at room temperature to allow for the annealing of HRP onto the target mimic sample/FITC probe. Finally, the whole SPCE was dipped into 0.4 mM 3,3′,5,5′-tetramethylbenzidine (TMB) and 0.4 mM H_2_O_2_ (Sigma) in a solution of a DEPC water mixture to elicit the signal that was measured by the chronoamperometry (CA) electrochemical method (Figure 1).

### 2.5. Electrochemical Equipment

The electrochemical detection equipment was manufactured by Metrohm (Herisau, Switzerland) Autolab, PGSTAT204, controlled by NOVA1.11, and analyzed using Origin 9.0 (Herisau, Switzerland). The counter electrode was made of platinum; the saturated calomel electrode was used as a reference electrode; the working electrode was the SPCE. We used two kinds of electrolytes for the experiments: one experiment used 0.1 M KCl and 5 mM Ferricyanide for the cyclic voltammetry analysis of the surface resistance, the other experiment applied 0.4 mM TMB and 0.4 mM H_2_O_2_ solution by CA to resolve target indication. The measurement setting was −200 mV against a saturated calomel electrode, the scan rate was 0.05 Vs^−1^, and the electroreduction current was performed using Origin 9.0 (Northampton, MA, USA). Furthermore, the chronoamperometric measurements were carried out to determine signals that were collected from the different FITC probe concentrations. Finally, the statistical analysis was performed using the GraphPad Prism 8 (GraphPad Software, La Jolla, CA, USA).

## 3. Results and Discussion

### 3.1. Optimization of Experimental Variables

In the current study, there are three variables that influence the detection of SARS-CoV-2 sequences, including the concentration of the biotinylated capture probe and the FITC probe hybridization concentrations, which affect the annealing to target sequences. Lastly, optimal biotin reactionary time was established for stability. Of course, there are other substrates and catalysts involved in this experiment to produce CV, CA, and LSV, including HRP, TMP, and H_2_O_2_ concentrations; however, as the establishment of optimal experimental values were mentioned in our previous experiment involving the same process, it is not discussed here [14]. Therefore, the concentrations used for this experiment were 0.4 mM for both TMP and H_2_O_2_, and 1 μg/mL for HRP.

#### 3.1.1. The Optimal Concentration of the Biotinylated Capture Probe

**Figure 2:**
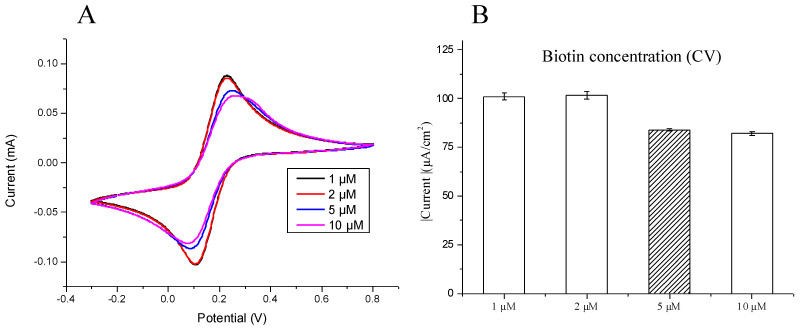
Generated electrochemical signals were observed from the modified surface of the screen-printed carbon electrode (SPCE) reacted with different concentrations of the biotinylated capture probe at room temperature for 1 h. We firstly confirmed by using (**A**) CV currents and (**B**) reduction potential value in different biotinylated capture probe concentrations. The optimal concentration was considered to be 5 μM. The bar chart also shows the triple examinations with confidence intervals.

To establish the optimal biotinylated capture probe concentration, a modified SPCE was tested against concentrations of 1, 2, 5, and 10 μM, and the results were observed using CV currents measured for reconfirmation. Furthermore, all the experiments were performed at least three times. As demonstrated in Figure 2A, there are higher cathodic and anodic peaks at lower biotinylated capture probe concentrations (1 and 2 μM) as compared with higher concentrations (5 and 10 μM), demonstrating lower current resistance and higher current density at low biotinylated capture probe concentrations, and vice versa with higher biotinylated capture probe concentrations. As previously mentioned, there is a reduction in current density with higher biotinylated capture probe concentrations; however, between 5 and 10 μM, there was no significant decline in current. Therefore, this indicated the appropriate saturation of biotinylated capture probes at 5 μM (Figure 2B), and we further demonstrated the most stable current density was 5 μM, which would be the optimal biotinylated capture probe concentration.

#### 3.1.2. The Optimal Reaction Time for the Biotinylated Capture Probe

**Figure 3:**
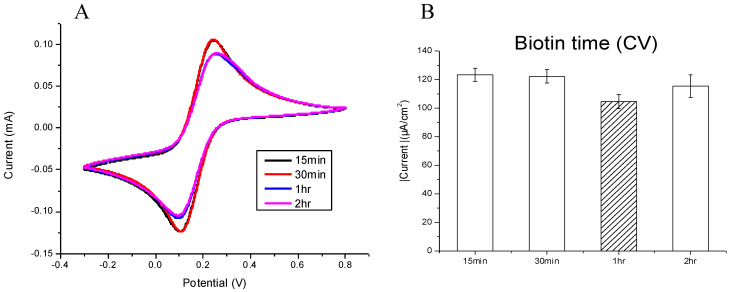
Observations of a range of time periods (15 min, 30 min, 1 h, and 2 h), the modified surface of SPCE reacted with the 5 μm biotinylated capture probe at room temperature, and the currents measured by (**A**) CV and (**B**) biotinylated capture probe. The 1 h reaction time showed the best reduction potential value. The bar chart also shows the triple examinations with confidence intervals.

Following the determination of the optimal testing concentration, different reaction periods were tested at 15 min, 30 min, 1 h, and 2 h while using 5 μm of biotinylated capture probe. Using the CV current, it is observed that the reactionary period has an inverse relationship to the current density measured at the anodic and cathodic peaks (Figure 3A). As compared with the current density of anodic peaks, there is a reduction in current density as the reactionary period increases. At 1 h we observe the most stable current density (Figure 3B).

### 3.2. The Optimal Concentration of the FITC Detector Probe

**Figure 4:**
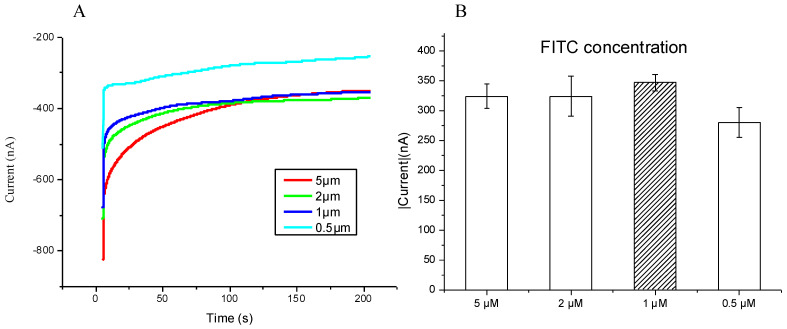
(**A**) Chronoamperometric diagram in different FITC probe concentrations; (**B**) the terminal current value in different concentrations of FITC probe by chronoamperometry, and 1 μm of FITC showed the best stability. The bar chart shows the triple examinations with confidence intervals.

The modified SPCE and 10 nM SARS-CoV-2 sequences were incubated at 55 °C. Then, 0.4 mM concentrations of both horseradish peroxidase and H_2_O_2_ were added along with the application of −0.3 V over 200 s. (Figure 4A), which demonstrated a direct correlation with the FITC concentration and current density, with low concentrations producing a low current density. Through repeat testing, 1 μm of FITC showed the best stability; hence, it was chosen to be the optimal concentration (Figure 4B).

### 3.3. Sensitivity and Reliability of the SARS-CoV-2 Biosensor

**Figure 5:**
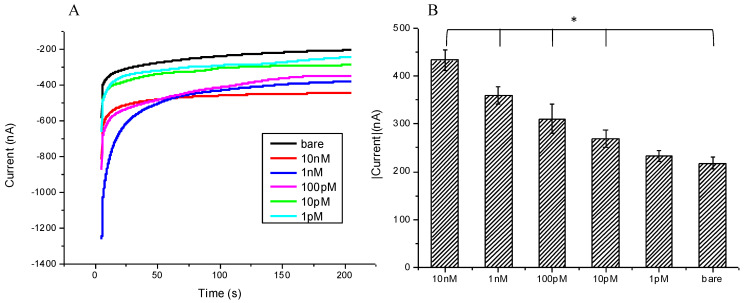
(**A**) Chronoamperometric diagram showing different concentrations of mimic SARS-CoV-2 target sequence were detected by the modified SPCE; (**B**) chronoamperometry to detect terminal current value, the currents decreased with the lower of concentrations of SARS-CoV-2 target sequence, the significant lowest concentration was 10 pM with *p*-value 0.0257. The bar chart also shows the triple examinations with confidence intervals. * meant p value < 0.005.

To challenge the traditional technique of RT-PCR, which currently is considered to be the best and most reliable method to confirm whether a person has a SARS-CoV-2 infection, herein, we pursue to establish the lowest detectable concentration by simply testing a modified SPCE against a concentration gradient of SARS-CoV-2 nucleotides, i.e., 10 nM, 1 nM, 100 pM, 10 pM, 1 pM, and a bare SPCE as control; using CA, we measured current responses. As seen in (Figure 5B), the current directly correlates to the concentration of the SARS-CoV-2 sequence, with a gradual reduction in current density as the concentration decreases. Notably, from the concentrations of 10 nM to 10 pM, all reactions showed significant *p*-values (0.0002, 0.0007, 0.0162, and 0.0257); however, at 1 pM and also 250 nA, the current was still measurable, but the *p*-values did not show significance (*p* = 0.27).

### 3.4. Stability and Repeatability of the SARS-CoV-2 Biosensor

**Figure 6:**
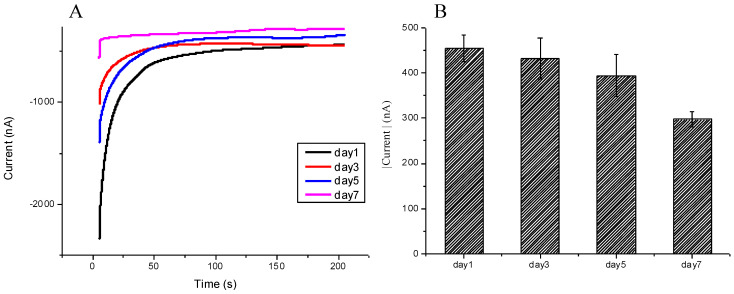
Demonstration of the stability of the modified SPCE after being stored in PBS solution for 1–7 days: (**A**) Chronoamperometric diagram demonstrated that the modified SPCE could be stored in PBS solution for at least 5 days and still presented reliable values of currents. (**B**) Days 1 to 5 might present reliable data; however, not day 7. The bar chart also shows the triple examinations with confidence intervals.

The storage of the modified SPCE has impacts on its ability to bind to the nucleic acid sequence and retain its sensitivity and reliability. This experiment tested the effects of the storage duration on the modified SPCE sensor. The SPCE was stored in a PBS solution at 4 °C for a number of days and then was tested against the 10 nM SARS-CoV-2 sequence; the results were measured with CV currents. From days 1 to 5, there were no notable differences in measured current; starting from day 7, there was a measurable decline in current density; the result differences from day 1 with significance of *p*-value 0.00029 (Figure 6A,B) indicated that the modified SPCE could be stored for at least 5 days, and still be ready to use.

### 3.5. Fitting Model Based on the Mimic Sample Obtained by the SARS-CoV-2 Biosensor

**Figure 7:**
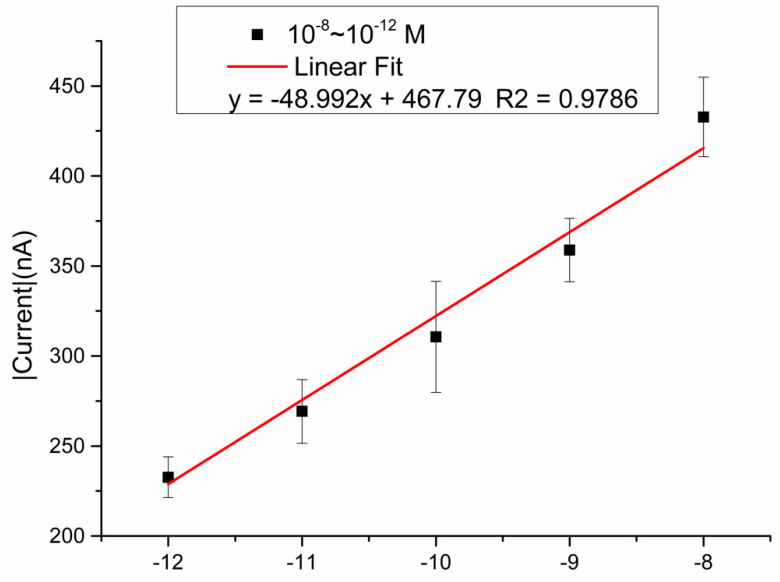
Demonstration of the mimic samples in different concentrations from 10^−12^ to 10^−8^ M, y = −48.992x + 467.79, and R squared is 0.9786.

Different concentrations of mimic samples (from −12 to −8 M) were detected via the SARS-CoV-2 biosensor, and triple detections were examined in each concentration; the results showed a linear fit curve, y = −48.992x + 467.79, R squared is 0.9786. (Figure 7).

### 3.6. Selectivity of the SARS-CoV-2 Biosensor

**Figure 8:**
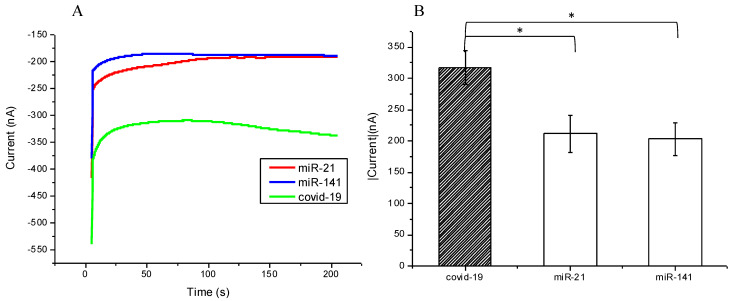
(**A**) Chronoamperometric diagram showing the modified SPCE interaction with different nucleotide sequences, but only binding with the COVID-19 (SARS-CoV-2) target sequence; (**B**) current values also presented significant differences for the COVID-19 sequence and miR-21 or miR-141 (*p*-value = 0.0218 and 0.0138, respectively). The bar chart also shows the triple examinations with confidence intervals. * meant *p* value < 0.005.

An array of samples was chosen for testing whether the biosensor would pick up background nucleic acids and produce false positives. Several single-nucleotide sequences were included, i.e., mimic SARS-CoV-2, microRNA-21, and microRNA-141, as well as a bare SPCE, which was used as a control. Then, via CA, we measured the current response. As expected, both microRNA-21 and microRNA-141 resulted in lower currents, similar to the bare SPCE at around 200 nA as compared with the SARS-CoV-2 sample nearly at 350 nA (Figure 8A), with negligible current variability (Figure 8B). The difference is appropriately significant (*p*-value 0.0218 and 0.0138) to demonstrate that random nucleic acid sequences do not anneal to the designed FITC detector probe and, therefore, do not interact with the modified SPCE, which proves its ability to effectively detect the desired sequences, in this case, the SARS-CoV-2 sequence.

### 3.7. Comparison of SARS-CoV-2 Biosensor and the Other Sensors

Herein, we further compared our current study results to the different performance parameters with other sensors, all with the same purpose of SARS-CoV-2 detection (Table 1). As shown in the table, our SPCE sensor also presented high sensitivity for detection.

## 4. Conclusions

The role and utility of biosensors in the field of rapid diagnostics have been emphasized now more than ever, especially in this time of the never-ending presence of coronavirus variants. This proof-of-concept study, using modifiable dual probes and detection via a modified SPCE, provides an opportunity to expand the field of electrochemical biosensors. Importantly, our biosensor demonstrates adequate sensitivity to SARS-CoV-2 without the need for amplification of nucleic acids and a rapid processing time of less than an hour. In addition, this biosensor has great potential since, due to its flexibility of probe design, it can be rapidly modified. According to the alterations of COVID-19 variant sequences reported by the WHO (World Health Organization) from time to time, we can easily and simply change the detecting probes, regardless of where the samples are derived, for example, serum, urine, saliva, or oropharyngeal swab; as long as the samples contain the RNA of the COVID-19 virus, detection should be possible. Indeed, tests on real samples would be necessary to evaluate the effectiveness of the proposed biosensor before promoting its commercialization. Nevertheless, our current study is proof of concept and provides prototype data. For real-world application or a clinical trial, a comprehensive plan would be required. Of course, there are multiple aspects to the biosensor that need to be addressed prior to commercialization, namely storage time and user interface. However, with the ingenuity of our probe, being its modifiability and a broad range of applications to other viruses and diseases involving nucleic acid sequences, we hoped to develop a biosensor that is able to adapt rapidly in times of need.

## Figures and Tables

**Table 1:** Comparison of our current study results and other similar SARS-CoV-2 detectors.

Sensors	Biomarkers	Limitation of Detection	References
Field-effect transistor (FET)	SARS-CoV-2 S protein	242 copies/mL	[1]
Glucometer	SARS-CoV-2 antigen	1 pM	[2]
Graphene field-effect transistor	COVID-19 spike protein S1	0.2 pM	[3]
Laser-engraved grapheme	SARS-CoV-2 antigen, antibodies	500 pg/mL	[4]
SPCE	SARS-CoV-2 specific single nucleotide RNA sequence	1 pM	Current study

## References

[1] Morales-Narváez E., Dincer C. (2020). The impact of biosensing in a pandemic outbreak: COVID-19. Biosens. Bioelectron..

[2] Chen L., Liu W., Zhang Q., Xu K., Ye G., Wu W., Sun Z., Liu F., Wu K., Zhong B. (2020). RNA based mNGS approach identifies a novel human coronavirus from two individual pneumonia cases in 2019 Wuhan outbreak. Emerg. Microbes Infect..

[3] Petrosillo N., Viceconte G., Ergonul O., Ippolito G., Petersen E. (2020). COVID-19, SARS and MERS: Are they closely related?. Clin. Microbiol. Infect..

[4] Lu R., Zhao X., Li J., Niu P., Yang B., Wu H., Wang W., Song H., Huang B., Zhu N. (2020). Genomic characterisation and epidemiology of 2019 novel coronavirus: Implications for virus origins and receptor binding. Lancet.

[5] Taleghani N., Taghipour F. (2020). Diagnosis of COVID-19 for controlling the pandemic: A review of the state-of-the-art. Biosens. Bioelectron..

[6] Dezfuli N.K., Adcock I.M., Montazami N., Mortaz E., Velayati A. (2020). Update on Immunology of COVID-19 Disease and Potential Strategy for Controlling. Tanaffos.

[7] Morehouse Z.P., Proctor C.M., Ryan G.L., Nash R.J. (2020). A novel two-step, direct-to-PCR method for virus detection off swabs using human coronavirus 229E. Virol. J..

[8] Corman V.M., Landt O., Kaiser M., Molenkamp R., Meijer A., Chu D.K., Bleicker T., Brünink S., Schneider J., Schmidt M.L. (2020). Detection of 2019 novel coronavirus (2019-nCoV) by real-time RT-PCR. Eurosurveillance.

[9] Sule W.F., Oluwavelu D.O. (2020). Real-time RT-PCR for COVID-19 Diagnosis: Challenges and Prospects. Pan. Afr. Med..

[10] Shen M., Zhou Y., Ye J., Al-Maskri A.A.A., Kang Y., Zeng S., Cai S. (2020). Recent advances and perspectives of nucleic acid detection for coronavirus. J. Pharm. Anal..

[11] Mojsoska B., Larsen S., Olsen D.A., Madsen J.S., Brandslund I., Alatraktchi F.A. (2021). Rapid SARS-CoV-2 Detection Using Electrochemical Immunosensor. Sensors.

[12] Mavrikou S., Moschopoulou G., Tsekouras V., Kintzios S. (2020). Development of a Portable, Ultra-Rapid and Ultra-Sensitive Cell-Based Biosensor for the Direct Detection of the SARS-CoV-2 S1 Spike Protein Antigen. Sensors.

[13] Chaibun T., Puenpa J., Ngamdee T., Boonapatcharoen N., Athamanolap P., O’Mullane A.P., Vongpunsawad S., Poovorawan Y., Lee S.Y., Lertanantawong B. (2021). Rapid electrochemical detection of coronavirus SARS-CoV-2. Nat. Commun..

[14] Leung W.H., Pang C.C., Pang S.N., Weng S.X., Lin Y.L., Chiou Y.E., Pang S.T., Weng W.H. (2021). High-Sensitivity Dual-Probe Detection of Urinary miR-141 in Cancer Patients via a Modified Screen-Printed Carbon Electrode-Based Electrochemical Biosensor. Sensors.

[15] Peng L., Liu J., Xu W., Luo Q., Chen D., Lei Z., Huang Z., Li X., Deng K., Lin B. (2020). SARS-CoV-2 can be detected in urine, blood, anal swabs, and oropharyngeal swabs specimens. J. Med. Virol..

